# Prevalence and Characteristics of Erectile Dysfunction in Obstructive Sleep Apnea Patients

**DOI:** 10.3389/fendo.2022.812974

**Published:** 2022-02-18

**Authors:** Chen Feng, Yan Yang, Lixiao Chen, Ruixiang Guo, Huayang Liu, Chaojie Li, Yan Wang, Pin Dong, Yanzhong Li

**Affiliations:** ^1^ Department of Otolaryngology Head and Neck Surgery, Shanghai General Hospital, Shanghai Jiaotong University School of Medicine, Shanghai, China; ^2^ Department of Otorhinolaryngology, Qilu Hospital, Shandong University Cheeloo College of Medicine, Jinan, China; ^3^ NHC Key Laboratory of Otorhinolaryngology (Shandong University), Jinan, China

**Keywords:** obstructive sleep apnea, erectile dysfunction, polysomnography, sex hormones, IIEF-5 questionnaire

## Abstract

**Background:**

Obstructive sleep apnea (OSA) is a common and severe social problem. Erectile dysfunction (ED) is an important health concern. The prevalence of OSA with ED is increasing, which significantly affects the quality of life and work efficiency of patients. However, the mechanism underlying the comorbidity of these two diseases remains unclear.

**Objectives:**

(1) Investigate the prevalence of OSA with ED; (2) analyze the correlation between OSA and ED; and (3) explore the treatment response to and possible mechanism of uvulapalatopharyngoplasty (UPPP) in patients with OSA and ED. This study aims to provide a theoretical basis for the clinical diagnosis and comprehensive treatment of OSA with ED and improve prevention and treatment strategies.

**Materials and Methods:**

In total, 135 subjects were enrolled in the study. Clinical data, polysomnography, the ESS score, Beck anxiety score, Beck depression score, IIEF-5 score and ASEX score were recorded before UPPP and 6 months after UPPP. Sex hormones were measured for all subjects using a Roche electrochemiluminescence analyzer.

**Result:**

The prevalence of OSA with ED was 64.52%, and the prevalence of severe OSA with ED was 73.02%. The prevalence of OSA with ED increased with age, BMI and apnea-hypopnea index (AHI) value. Among polysomnography indicators, minimum oxygen saturation and average oxygen saturation may predict the occurrence of OSA with ED. Improving the patient’s anxiety and depression is very important for treating OSA with ED. Sex hormone levels were not significantly correlated with the occurrence of OSA with ED.

**Conclusion:**

ED is a common symptom of OSA patients. This study showed that sex hormone levels in OSA patients with ED were not significantly correlated with the condition, but further investigation of this relationship is worthwhile. It is recommended that the free and combined types of sex hormones be further distinguished during testing because the free type is the active form. UPPP surgical treatment is effective for OSA with ED, and its possible mechanism is protection of the peripheral nerves of the sex organs by improving nighttime hypoxia and arousal.

## Introduction

Obstructive sleep apnea (OSA) is a serious health hazard that requires long-term, multidisciplinary therapy and has a prevalence of 9% to 38% in the general population (1, 2). Previous studies have shown that OSA is a significant independent factor for hypertension, diabetes, coronary heart disease and other diseases (3–6). OSA has become a common and serious social problem that significantly affects the quality of life and work efficiency of patients, especially those who are overweight (7). Snoring, excessive daytime sleepiness, inattention, and erectile dysfunction (ED) are common comorbidities in OSA patients (8, 9).

Due to recurrent snoring, apnea and microarousal, OSA patients may have the following pathophysiological changes (10). First, repeated awakening at night can significantly reduce non-rapid eye movement (NREM) sleep and rapid eye movement (REM) sleep, resulting in sleep structure disorder, reduced sleep efficiency, daytime drowsiness, fatigue, memory loss and hormone secretion disorder. Second, chronic intermittent hypoxia can cause increased catecholamine secretion and vascular endothelial injury, leading to hypertension and atherosclerosis. Finally, decreased blood oxygen saturation is also closely related to arrhythmia, and increased erythropoietin levels can lead to increases in hemoglobin levels, the number of red blood cells, and platelet activity and reduced fibrinolytic activity and then induce coronary heart disease and cerebral thrombosis. The quality of REM sleep in OSA patients is generally worse than that of NREM sleep (11, 12). Series (12) has shown that in patients with OSA, the decrease in blood oxygen saturation (SaO_2_) during REM sleep is greater than that during NREM sleep. Moreover, Findley (11) found that the sleep apnea duration during REM sleep is longer than that during NREM sleep, and hypoxemia is more severe. And Shi’s study (13) showed sleep duration for men and women may be independent predictors of conception.

According to the National Institutes of Health Consensus meeting, ED is defined as a persistent inability to achieve or maintain an erection or sustain an adequate sexual relationship (14). Erection is an event involving the interaction of the psychological, neurological, endocrine and vascular systems. It is estimated that by 2025, 300 million men worldwide will be living with ED (15). Guilleminault (16) was the first to study the relationship between erectile function and OSA and showed a higher prevalence of ED in patients with severe OSA (48%). Subsequent epidemiological and clinical studies have supported the conclusion that OSA is associated with ED (17–22). Kellesarian (23) reported in the review that the prevalence of OSA patients with ED is between 40.9% and 80% and that the risk of ED in patients without OSA is significantly lower than that in OSA patients. Furthermore, Chen’s study (19) showed that the prevalence of ED in OSA patients was 9.44 times higher than that in non-OSA patients and that OSA was an independent risk factor for the development of ED. Smith (24) reported that OSA with ED is closely related to patients’ psychological states, such as depression and anxiety. Moreover, studies have shown that continuous positive airway pressure ventilation (CPAP) (17, 25–27) and oral orthotics (17) used to treat OSA can improve ED. However, Stannek (21) reported that the severity of OSA may not be related to the severity of ED. A correlation analysis between erectile function and polysomnography (PSG) results may suggest that factors such as decreased REM sleep can lead to peripheral nerve damage in the sexual organs in patients, resulting in ED (28). In the sleep state, the REM period is usually accompanied by erection, and most of the REM period occurs in the morning, so early morning erection is a common phenomenon (29). And Chen’s study (30) found that sleep deprivation or oversleeping can also affect sperm quality. Notably, Andersen (20) reported that reduced REM sleep and increased arousal negatively affected erectile function in male rats. Chen (31) reported male sleep quality and duration may impact male fertility, an important consequence of poor semen quality.

The major factor associated with ED may be endothelial dysfunction, including reduced nitric oxide (NO) production and elevated endothelin levels (32, 33). NO, the most important mediator of penis swelling, plays a key role in the physiological process of erection by stimulating blood vessel dilation, increasing blood flow to the cavernous body and promoting smooth muscle relaxation (34–36), but the molecular levels of NO in OSA have been less studied. Studies have shown that both hypoxia and enhanced oxygen metabolism can stimulate the transcription of pre-endothelin, forming endothelin, the strongest vasoconstrictive substance (32, 33) known to date. Its long-lasting effect is an endogenous long-acting vasoconstrictive regulator that can cause the contraction of spongy smooth muscle cells (37), which has been verified in the experimental environment and OSA patients (38). In addition, there are other nonvascular mechanisms that can explain the mechanism of ED in OSA patients, including changes in hormone levels, as follows: hypothalamic-pituitary-gonadal (HPG) axis (39–41) regulation disorder leading to changes in hormone levels; neurological mechanisms, such as hypothalamic-pituitary-adrenal (HPA) axis (42) regulation disorders and neurological dysfunction; and psychological mechanisms, such as reduced libido and excessive fatigue (43). Although many studies have been conducted on OSA-related endocrine levels, few studies have investigated the HPG axis regulation mechanism in OSA patients (41). There are still many unclear mechanisms in the relationship between OSA and ED, and there are few prospective studies and basic studies with large sample sizes, so no consistent conclusion has been reached. In this study, the clinical data of OSA patients were used to explore the correlation between OSA and ED.

## Materials and Methods

### Research Process

**Figure 1:**
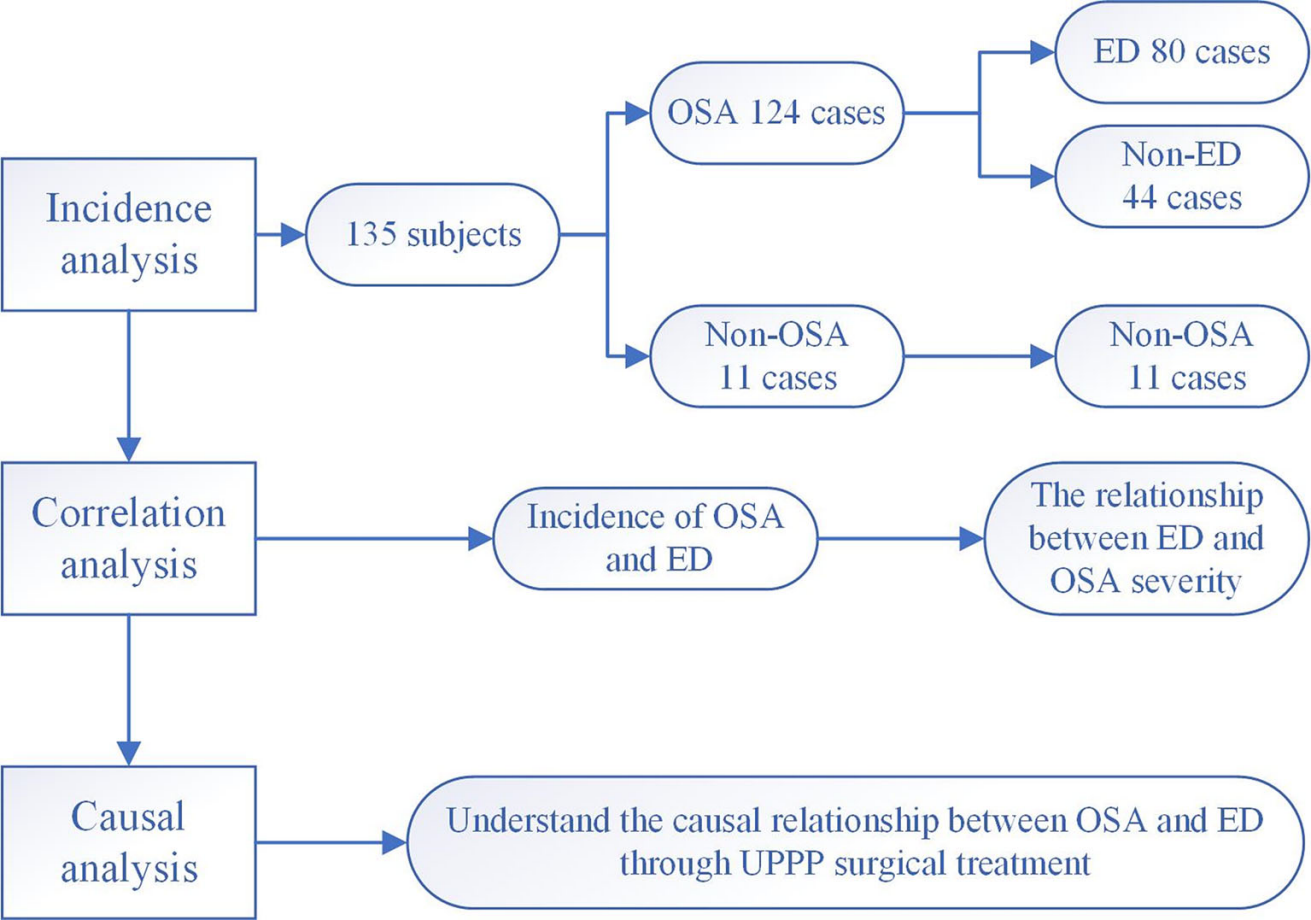
Research flow chart. 135 male subjects with relevant clinical data were included to explore the mechanism underlying the comorbidity of OSA and ED.

First, the prevalence the cooccurrence of ED and OSA was investigated. Second, we explored the clinical characteristics of OSA and ED. Finally, the treatment response was assessed. The flow chart is shown in **Figure 1**.

### Source of Cases

The subjects of the study were male patients had completed PSG for OSA at the sleep monitoring center from October 2019 to December 2020. The patients reported habitual snoring and daytime sleepiness, with or without nocturnal apnea. The patients were informed of the study plan, and the subjects voluntarily signed the informed consent form following the guidelines of the National Ethics Regulation Committee and in accordance with the Declaration of Helsinki.

### Exclusion Criteria

To ensure scientific integrity, the following conditions were excluded: patients older than 60 years and younger than 18 years; patients taking medications that may affect erectile function, including cardiovascular drugs (beta-blockers, clonidine, diuretics and digoxin), antidepressants (tricyclic antidepressants and selective serotonin reuptake inhibitors), antipsychotics, antiepileptics, 5-reductase inhibitors, sedatives, cimetidine and opioids; patients with chronic oxygen-deficient respiratory diseases such as chronic obstructive pulmonary disease (COPD); and patients with a history of epilepsy, mental illness, and other disorders that cause erectile dysfunction.

### Sample Size Calculation

We used formulas to estimate the sample size of the research. According to the literature (23) the prevalence of OSA with ED was the lowest 41.0%. We set the probability of the first type of error α=0.05, and used the software EXCEL to calculate the function: Z_α/2_=NORM.S.INV(1-α/2) =1.960. β was the probability of making the second type of error. The greater the test power required by the experimental research, the larger the sample size required. It was generally assumed that β=0.10, and the statistical power 1-β was 90%. The value range of δ was (0.25S, 0.50S), and we assumed that the allowable error δ=0.15. Then, we used the software EXCEL to calculate the function: sample size n = ((NORM.S.INV(1-α/2))/(ASIN(δ/(p*(1-p)) ^0.5)))^2. That is, when the sample size was 40.0, it could meet the inspection requirements. Therefore, our sample size was in line with the sample size estimate.

### General Information

The enrollees were surveyed about general demographic data, including basic information about age, height, weight, blood pressure, smoking status, drinking status, neck circumference, waist circumference, and hip circumference, and the body mass index (BMI) value was calculated.

The BMI value adopts the Chinese adult standard (44), namely, a BMI value less than 18.5 means low body weight, a BMI value between 18.5 and 24.0 means normal weight, a BMI value between 24.0 and 28.0 means overweight, and a BMI value greater than or equal to 28.0 means obesity. To ensure the authenticity of the information, information collection and questionnaires were conducted anonymously, and specialized clinical technicians explained and verified the contents of the questionnaires.

### PSG Data Acquisition and Interpretation Criteria

Overnight PSG includes encephalogram (EEG), electrooculogram (EOG), electromyography (EMG) of the chin muscles, electrocardiogram (ECG), nasal air measurement using pressure sensors, oral air measured with thermistors, chest and abdomen movements, snoring analysis, sleep posture, leg movements and finger pulse oxygen saturation (SO_2_) detection. OSA was categorized as mild OSA (5-15 times/h), moderate OSA (15-30 times/h) and severe OSA (more than 30 times/h) according to the apnea-hypopnea index (AHI) value. Manual data analysis was performed by two experienced sleep technicians according to the scoring standards in the diagnostic and treatment guidelines of the American Academy of Sleep Medicine (AASM) (45).

### Assessment of Erectile Function

The International Index of Erectile Function (IIEF-5) was used as the main diagnostic basis for erectile dysfunction and includes five aspects: erectile function, orgasm function, libido function, sexual satisfaction and overall satisfaction. An IIEF-5 score less than or equal to 7 indicates severe erectile dysfunction, between 8 and 11 indicates moderate erectile dysfunction, between 12 and 21 indicates mild erectile dysfunction, and greater than or equal to 22 indicates normal erectile function (46).

The Arizona Sexual Experience Scale (ASEX) was used as an auxiliary measure to assess sexual function. There are 5 questions on this scale, and each question is rated as 1-6 points according to sexual function hyperactivity and sexual function depression. The evaluation areas include sexual drive, sexual vigilance, penile erection, orgasm ability and sexual satisfaction. A comprehensive assessment of the patient’s sexual function was carried out. Three or more items on the ASEX scale with a score greater than 4 points or any single item scoring greater than 5 or a total score greater than 19 points is indicative of sexual dysfunction (47). The contents of and instructions for the scale were introduced by specialized clinical technicians according to unified guidance, and subjects were given 10-20 minutes to complete it.

### Sleep Quality Assessment

The Epworth Sleepiness Scale (ESS) was used to assess daytime sleepiness. There are 8 questions in the scale, and the probability of dozing in each question is “never”, “mild”, “moderate” and “severe”. The scores are 0, 1, 2 and 3, with the highest total score being 24 points. An ESS score greater than 10 is classified as daytime sleepiness (48). The contents of and instructions for the scale were introduced by specialized clinical technicians according to unified guidance, and the time allotted was 5-10 minutes.

### Assessment of Anxiety and Depression Symptoms

The Beck Anxiety Inventory (BAI) and the Beck Depression Inventory (BDI-II) were used to evaluate the subjective anxiety and depression status of the study subjects. There are 21 test dimensions in the BAI, and the four expressions correspond to the numbers 1, 2, 3, and 4. Number 1 represents nondisturbing anxiety, from milder to stronger, and number 4 represents symptoms of disturbing anxiety that can only barely be tolerated. The sum of the numbers is the evaluation result, and a score greater than or equal to 45 indicates that the patient is in an anxious state (49).

There are also 21 test dimensions in the BDI-II, which correspond to the numbers 0, 1, 2, and 3. The total score of the 21 items is the final test score. A total score of 0-13 means no depression, 14-19 means mild depression, 20-28 means moderate depression, and 29-63 means severe depression (50). The contents of and instructions for the scale were introduced by specialized clinical technicians according to the unified guidance, and the subjects were given 10-20 minutes for completion.

### Peripheral Blood Collection for Assessment of Sex Hormones

At approximately 7:00 in the morning, approximately 3 ml of blood was drawn from the antecubital vein of each enrolled patient into a tube containing no anticoagulant, and the tubes were placed in a water bath for 10 minutes. The temperature was controlled at approximately 37°C. After removal, each sample was centrifuged at 2000 r/min for 10 minutes and then stored at -20°C. Tests for six sex hormones—follicle-stimulating hormone (FSH), luteinizing hormone (LH), testosterone (TEST), estradiol (E2), progesterone (PROG) and prolactin (PRL)—were performed by a Roche electrochemiluminescence analyzer.

### OSA-Related Treatment

After the diagnosis of OSA in male patients with ED symptoms, uvulopalatopharyngoplasty (UPPP) was performed, and after treatment, the PSG and IIEF-5 scores of the patients were collected as described above.

### Statistical Methods

SPSS 24.0, Prism 9 and Excel were used to process the data. Normally distributed measurement data are expressed as the mean ± standard deviation, and nonnormally distributed measurement data are expressed as the median (interquartile range) [M (Q25~Q75)]. For normally distributed measurement data, an independent-sample t-test or Pearson’s chi-square test was used for comparisons between the two groups, and one-sample ANOVA was used for comparisons among three groups. For nonnormally distributed measurement data, the Mann-Whitney U test was used for comparisons between two groups, and the Kruskal-Wallis H test was used for comparisons among three groups. Spearman’s correlation analysis was used to test the correlation between nonnormally distributed measurement data, and Pearson’s correlation analysis was used to test the correlation between normally distributed measurement data. Variables that were found to be significant in univariate analysis were included in multivariate analysis, and binary logistic regression analysis was performed. The receiver operating characteristic (ROC) curve was used to evaluate the AUC (area under the curve) of the relevant factors and then evaluate the performance of each factor as a criterion. Count data were used to calculate the composition ratio using the χ^2^ test. For normally distributed measurement data, the paired-samples t-test was used to compare the differences before and after treatment. The inspection level was set at α=0.05.

## Results

### General Conditions

#### Comparison of Basic Data

**Figure 2:**
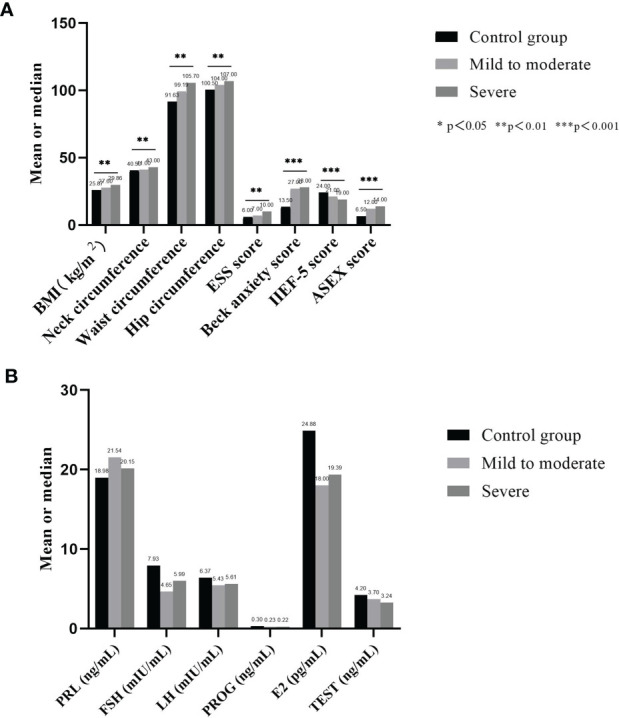
Comparison of basic data and hormone secretion levels of each group. **(A)** shows that among the non-OSA subjects, patients with mild to moderate OSA, and patients with severe OSA, the BMI, neck circumference, waist circumference, hip circumference, ESS score, Beck anxiety score and ASEX score showed a significant increase, while the IIEF-5 score significantly decreased; **(B)** shows that no significant difference was found in the secretion levels of the six serum sex hormones among the three groups.

There were 30 male subjects with incomplete questionnaires, and 5 male subjects met the exclusion criteria. Ultimately, 135 male subjects with relevant clinical data were included. The median age was 37 years old, BMI was 28.55 ± 4.12 kg/m2, and AHI value was 35.89 ± 23.81 times/h. There were 11 non-OSA subjects, 61 patients with mild to moderate OSA and 63 patients with severe OSA. **Table 1** summarizes the demographic profile and related clinical phenotypes of the included population. The results show that among the non-OSA subjects, patients with mild to moderate OSA, and patients with severe OSA, the BMI (p=0.001), neck circumference (p=0.005), waist circumference (p=0.001), hip circumference (p=0.006), ESS score (p=0.003), Beck anxiety score (p<0.001) and ASEX score (p<0.001) showed a significant increase, while the IIEF-5 score (p<0.001) significantly decreased (**Figure 2A** and **Table 1**).

**Table 1:** Basic demographics and related clinical phenotypes of the population included in the study.

Clinical Phenotype	Total (n=135)	OSA Patients			Non-OSA Subjects (n=11)	P
		Total (n=124)	Mild to Moderate (n=61)	Severe (n=63)		
Age	37.00 (31.00~44.00)	37.00 (31.00~44.00)	36.00 (30.50~45.00)	38.00 (32.00~43.00)	41.00 (29.00~47.00)	0.906
Smoking status (%)	60 (44.44)	51 (41.13)	21 (34.43)	30 (47.62)	8 (72.73)	0.064
Alcohol consumption (%)	90 (66.67)	83 (66.94)	41 (67.21)	42 (66.67)	6 (54.55)	0.537
BMI (kg/m^2^)	28.55 ± 4.12	28.74 ± 4.09	27.60 ± 4.26	29.86 ± 3.61	25.87 ± 3.72	**0.001**
Neck circumference (cm)	42.00 (39.75~45.00)	42.00 (40.00~45.00)	41.00 (38.00~43.75)	43.00 (40.00~45.00)	40.50 (36.50~42.75)	**0.005**
Waist circumference (cm)	102.31 ± 11.96	103.15 ± 11.71	99.19 ± 12.18	105.70 ± 10.74	91.63 ± 10.36	**0.001**
Hip circumference (cm)	105.00 (101.00~111.00)	106.00 (102.00~111.00)	104.00 (99.13~109.75)	107.00 (103.00~112.25)	100.50 (94.75~104.50)	**0.006**
Systolic blood pressure (mmHg)	132.00 (124.50~140.00)	132.00 (125.75~140.00)	132.00 (123.00~140.00)	133.00 (128.00~140.00)	128.00 (109.00~138.00)	0.320
Diastolic blood pressure (mmHg)	83.80 ± 9.44	83.99 ± 9.45	83.42 ± 9.59	84.38 ± 9.41	80.86 ± 9.55	0.614
ESS score	9.00 (6.00~13.00)	9.00 (6.00~13.00)	7.00 (4.00~11.00)	10.00 (6.00~15.00)	6.00 (4.00~9.25)	**0.003**
Beck anxiety score	27.00 (22.00~33.00)	28.00 (22.00~33.75)	27.00 (22.50~32.50)	28.00 (24.00~34.00)	13.50 (12.00~15.75)	**<0.001**
Beck depression score	7.00 (3.75~11.25)	7.00 (4.00~12.00)	7.00 (3.50~11.00)	8.00 (4.00~13.00)	5.00 (0.75~7.25)	0.137
IIEF-5 score	20.00 (18.00~23.00)	20.00 (17.25~22.00)	21.00 (19.00~23.00)	19.00 (17.00~22.00)	24.00 (22.75~25.00)	**<0.001**
ASEX score	13.00 (11.00~15.00)	13.00 (11.00~15.00)	12.00 (11.00~14.00)	14.00 (12.00~16.00)	6.50 (5.00~9.25)	**<0.001**

The meaning of the bold values is p<0.05.

#### Comparison of Serum Sex Hormone Levels

**Table 2** summarizes the sex hormone test data of the included population. The results show that no significant difference was found in the secretion levels of the six serum sex hormones among the three groups: PRL (p=0.728), FSH (p= 0.062), LH (p= 0.294), PROG (p=0.821), E2 (p= 0.686) and TEST (p= 0.056) (**Figure 2B**).

**Table 2:** Sex hormone test data of the population included in the study.

Sex Hormones	Total (n=135)	OSA patients			Non-OSA Subjects (n=11)	P
		Total (n=124)	Mild to moderate (n=61)	Severe (n=63)		
PRL (ng/mL)	18.88 (14.04~24.97)	18.88 (14.09~24.38)	18.81 (15.07~28.31)	19.29 (13.99~22.79)	19.55 (11.43~26.59)	0.728
FSH (mIU/mL)	5.10 (3.80~6.84)	5.00 (3.79~6.68)	4.76 (2.98~5.73)	5.26 (4.04~6.86)	7.30 (3.73~12.46)	0.062
LH (mIU/mL)	5.28 (3.85~6.76)	5.06 (3.83~6.69)	4.38 (3.82~6.90)	5.20 (3.83~6.28)	6.64 (5.36~7.47)	0.294
PROG (ng/mL)	0.20 (0.13~0.30)	0.20 (0.13~0.30)	0.21 (0.11~0.30)	0.20 (0.13~0.30)	0.22 (0.14~0.54)	0.821
E2 (pg/mL)	18.76 (12.82~22.95)	18.89 (12.89~22.79)	18.14 (15.46~20.37)	19.67 (11.86~23.82)	17.56 (10.26~44.79)	0.686
TEST (ng/mL)	3.42 ± 1.14	3.36 ± 1.15	3.70 ± 1.17	3.24 ± 1.12	4.20 ± 0.81	0.056

FSH, Follicle stimulating hormone; LH, luteinizing hormone; and TEST, testosterone; E2, estradiol; PROG, progesterone; and PRL, prolactin.

### Prevalence of OSA and ED

There were 124 patients with confirmed OSA and 63 patients with severe OSA. The prevalence of OSA was 91.85% (124/135), with “snoring during sleep” as the chief complaint.

#### Prevalence of ED and OSA With ED

**Figure S1** summarizes the IIEF-5 score of the included population. The results show that a total of 80 ED patients were included in the population, with an prevalence of 59.26% (80/135), and among these subjects, the prevalence of mild ED was 56.30% (76/135), the prevalence of moderate ED was 0.74% (1/135), and the prevalence of severe ED was 2.22% (3/135). There were a total of 80 patients with OSA with ED, with an prevalence of 64.52% (80/124), among whom the prevalence of mild ED was 61.29% (76/124), the prevalence of moderate ED was 0.81% (1/124), and the prevalence of severe ED was 2.42% (3/124). For the 34 patients with mild to moderate OSA with ED, the prevalence rate was 55.74% (34/61), of which the prevalence of mild ED was 52.46% (32/61), the prevalence of moderate ED was 1.64% (1/61), and the prevalence of severe ED was 1.64% (1/61). A total of 46 patients with severe OSA with ED had an prevalence of 73.02% (46/63), among whom the prevalence of mild ED was 69.84% (44/63), the prevalence of severe ED was 3.17% (2/63), and the prevalence of moderate ED was 0 (statistical level). Mild to moderate OSA patients and severe OSA patients with OSA with ED were mainly characterized by mild ED.

#### Prevalence of OSA With ED in Different AHI Groups

**Figure 3:**
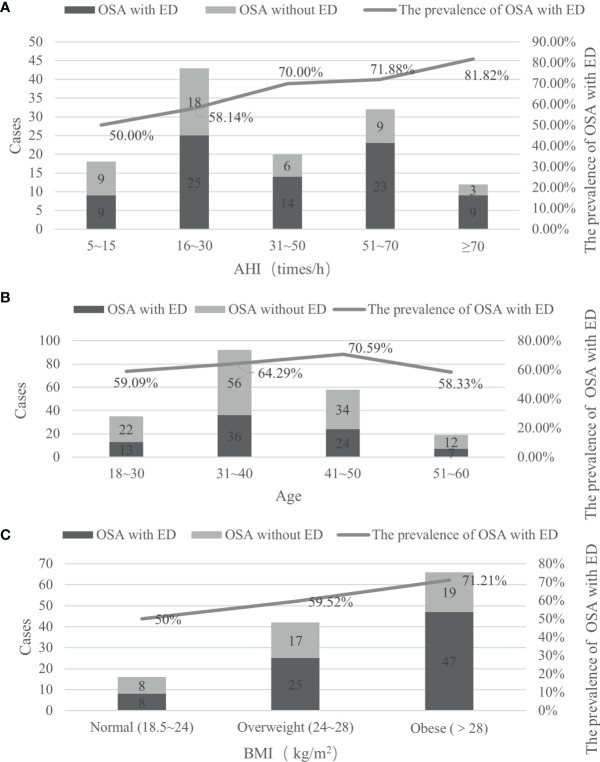
The prevalence of OSA with ED in different AHI, age and BMI groups. **(A)** It was found that with the increase in AHI values, the prevalence of OSA with ED gradually increased; **(B)** the prevalence rate gradually increased with age before the age of 50; **(C)** Among OSA patients, as BMI increased, the prevalence of concurrent ED increased.

To clarify the relationship between AHI values and the prevalence of OSA with ED, the patients in different AHI groups were analyzed, and it was found that with the increase in AHI values, the prevalence of OSA with ED gradually increased. When the AHI score was greater than or equal to 70 times/h, the prevalence OSA with ED reached a maximum of 81.82%; when the AHI score was 51 to 70 times/h, the prevalence was 71.88%; when the AHI score was 31 to 50 times/h, the prevalence was 70.00%; when the AHI was 16 to 30 times/h, the prevalence was 58.14%; and when the AHI score was 5 to 15 times/h, the minimum prevalence rate was 50.00% (**Figure 3A**).

The prevalence of OSA with ED increased with the increase in severity of OSA (OR=2.818, p<0.001); that is, for every increase in the AHI value of OSA patients by 1 time/h, the prevalence of OSA with ED increased by 2.818 times. The odds of patients with mild to moderate OSA having concurrent ED was OR=2.259, p=0.001; that is, every time the AHI value of patients with mild to moderate OSA increased by 1 time/h, the prevalence of ED increased by 2.259 times. The odds of patients with severe OSA having ED was OR=3.706, p<0.001; that is, every time the AHI value of patients with severe OSA increased by 1 time/h, the prevalence of ED increased by 3.706 times (**Table 3**).

**Table 3:** The prevalence of ED in patients with OSA of different types of clinical data.

Types of Clinical Data	Grouping	OSA with ED	OSA Without ED	P	OR (95%CI)	OR (95%CI)
Different severity groups	Non-OSA subjects	0	11	–	–	–
	OSA patients	80	44	<0.001	2.818 (2.223-3.573)	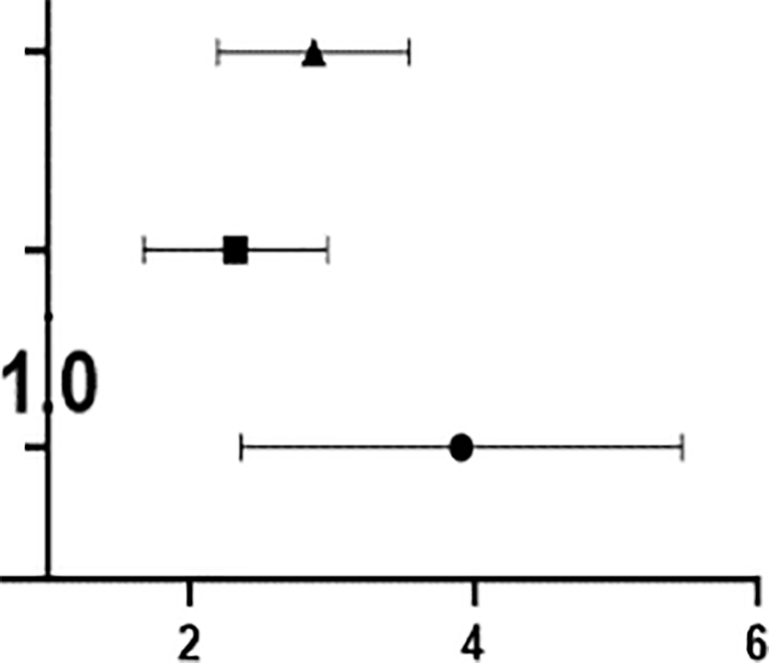
	Mild to moderate OSA	34	27	0.001	2.259 (1.705-2.994)	
	Severe OSA	46	17	<0.001	3.706 (2.469-5.563)		
Different age groups	Control	0	11		–	–
	18~30	13	9	0.001	2.444 (1.479-4.039)	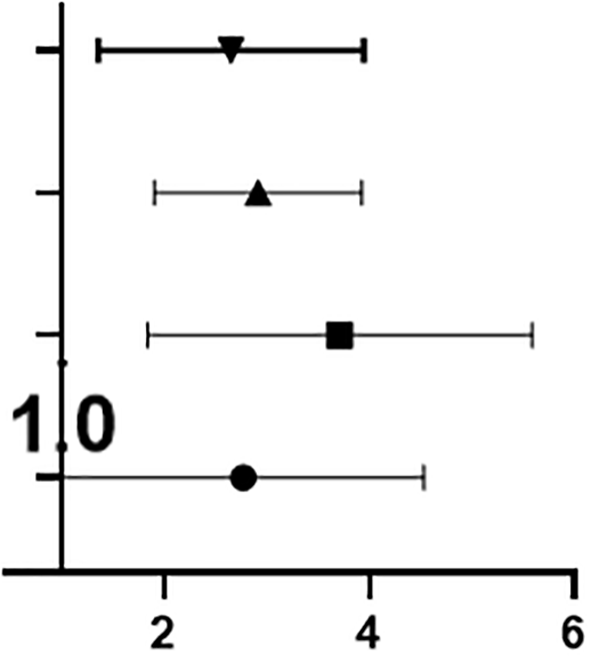
	31~40	36	20	<0.001	2.800 (1.970-3.979)	
	41~50	24	10	<0.001	3.400 (2.020-5.723)	
	51~60	7	5	0.002	2.400 (1.229-4.688)	
Different BMI groups	Control	0	11	–	–	–
	Normal (18.5~24)	8	8	0.005	2.000( 1.225-3.265)	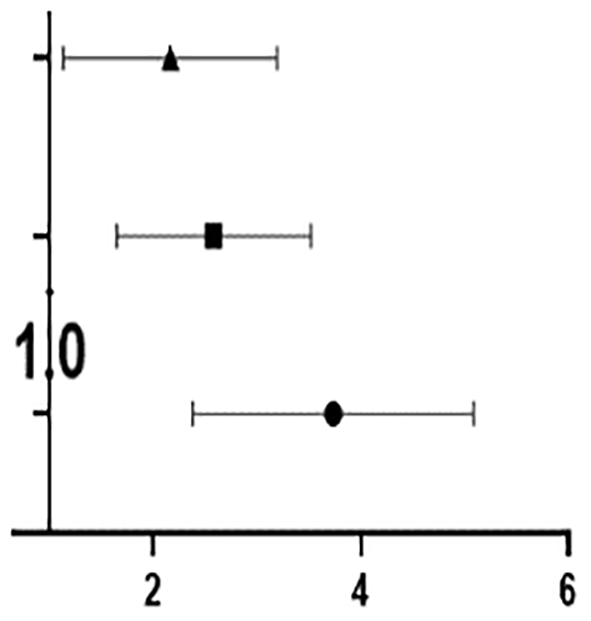
	Overweight (24~28)	25	17	<0.001	2.471 (1.712-3.565)	
	Obese (>28)	47	19	<0.001	3.474 (2.377-5.077)	

#### Prevalence of OSA With ED in Different Age Groups

To clarify the relationship between age and the prevalence of OSA with ED, an analysis of patients in different age groups revealed that the prevalence of OSA with ED was 64.52% (80/124), and the highest prevalence rates were 70.59% in the 41-50 group, 64.29% in the 31-40 group, 59.09% in the 18-30 group, and 58.33% in the 51-60 group, indicating that the prevalence rate gradually increased with age before the age of 50. In the severe OSA population, the prevalence of OSA with ED was 74.60% (47/63); the highest prevalence rates were 85.71% in the 18-30-year-old group, followed by 80.00% in the 51-60-year-old group and 71.88% in the 31-40-year-old group, and the lowest prevalence was 68.42% in the 41-50-year-old group (**Figure 3B**).

The prevalence of OSA with ED increased with age (OR=2.444, p=0.001), which means that for every 1-year increase in age of OSA patients, the prevalence of ED increased by 2.444 times. The OR of the 31- to 40-year-old group was 2.800, p <0.001; that is, the prevalence of OSA with ED increased by 2.800 times when the age of OSA patients increased by 1-year in this group. The OR of the 41- to 50-year-old group was 3.400, p <0.001; that is, for every 1-year increase in age of OSA patients in this group, the prevalence of OSA with ED increased by 3.400 times. Finally, the OR of the 51- to 60-year-old group was 2.400, p = 0.002; that is, for every 1-year increase in the age among the OSA patients, the prevalence of ED increased by 2.400 times (**Table 3**).

#### Prevalence of OSA With ED in Different BMI Groups

To clarify the relationship between BMI and the prevalence of OSA with ED, an analysis of patients in different BMI groups was carried out. Among OSA patients, as BMI increased, the prevalence of concurrent ED increased, and the prevalence in the obesity group was as high as 71.21%, followed by 59.52% in the overweight group and 50.00% in the normal weight group. The same trend was observed in the severe OSA population. The obesity group had the highest prevalence rate of 76.92%, followed by the overweight group (68.18%), and the normal weight group had the lowest prevalence rate (50.00%) (**Figure 3C**).

The prevalence of ED among OSA patients increased as the BMI value increased. The OR of the normal weight group was 2.000, p = 0.005; that is, for every increase in BMI value of 1 kg/m^2^ among OSA patients, the prevalence of ED increased by 2.000. The OR of the overweight group was 2.471, p <0.001; that is, for every increase of 1 kg/m^2^ in BMI among OSA patients, the prevalence of ED increased by 2.471 times. The OR of the obese group was 3.474, p<0.001; that is, for every increase of 1 kg/m^2^ in BMI among OSA patients, the prevalence of ED increased by 3.474 times (**Table 3**).

### Differences in Population Characteristics Between OSA Patients With ED and OSA Patients Without ED

#### Basic Demographics and Related Clinical Phenotypes of OSA With ED and OSA Without ED

**Figure 4:**
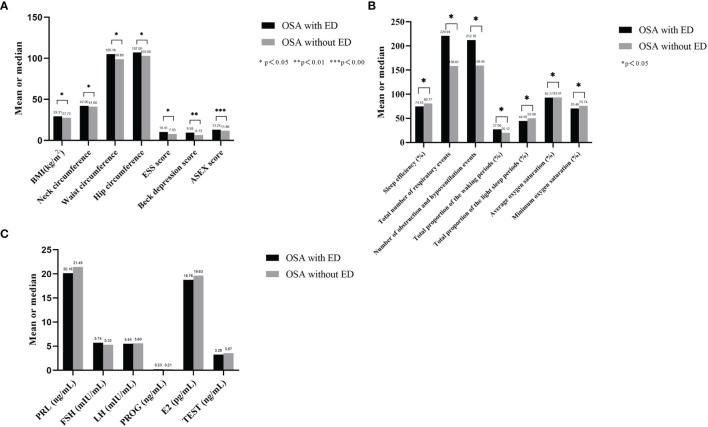
Basic related clinical phenotypes, PSG data and sex hormone secretion of OSA with ED and without ED. **(A)** BMI, neck circumference, waist circumference, hip circumference, ESS score, Beck depression score and ASEX score were higher in the OSA with ED group than in the OSA without ED group; **(B)** Sleep efficiency, average oxygen saturation and minimum oxygen saturation were significantly lower in the OSA with ED group than in the OSA without ED group. Moreover, the total number of respiratory events, number of obstruction and hypopnea events, total proportion of the waking periods and total proportion of the light sleep periods were significantly higher in the OSA with ED group than in the OSA without ED group; **(C)** The secretion levels of PRL, FSH, LH, PROG and TEST were not significantly different between the groups.

A comparison of OSA patients with ED and OSA patients without ED showed that BMI (p=0.039), neck circumference (p=0.046), waist circumference (p=0.011), hip circumference (p=0.025), ESS score (p=0.040), Beck depression score (p=0.011) and ASEX score (p<0.001) were higher in the OSA with ED group than in the OSA without ED group (**Table 4** and **Figure 4A**).

**Table 4:** Basic demographics and related clinical phenotypes of OSA with ED and without ED.

Clinical Phenotype	OSA with ED (n=80)	OSA without ED (n=44)	P
Age	36.50 (31.00~44.75)	37.00 (31.25~43.75)	0.973
Smoking status (%)	38 (47.50)	13 (29.55)	0.059
Alcohol consumption (%)	56 (70.00)	27 (61.36)	0.420
BMI (kg/m^2^)	29.31 ± 4.08	27.72 ± 3.94	**0.039**
Neck circumference (cm)	42.00 (40.00~45.00)	41.00 (38.00~43.00)	**0.046**
Waist circumference (cm)	105.18 ± 10.86	98.89 ± 12.44	**0.011**
Hip circumference (cm)	107.00 (103.00~112.00)	103.00 (99.25~110.50)	**0.025**
Systolic blood pressure (mmHg)	132.00 (128.00~140.25)	132.50 (120.50~140.00)	0.616
Diastolic blood pressure (mmHg)	85.23 ± 9.14	81.58 ± 9.70	0.060
ESS score	10.00 (6.00~15.00)	8.00 (5.25~11.00)	**0.040**
Beck anxiety score	28.00 (23.00~35.00)	27.00 (23.00~31.00)	0.367
Beck depression score	8.00 (5.00~13.00)	6.00 (2.25~8.00)	**0.011**
ASEX score	14.00 (12.00~16.00)	12.00 (10.00~13.00)	**<0.001**
AHI (times/h)	41.10 (16.90~60.23)	24.25 (17.45~50.40)	0.102
Sleep efficiency (%)	77.45 (67.00~87.68)	84.90 (74.63~91.23)	**0.036**
Average pause and hypopnea time (s)	22.00 (21.00~24.00)	22.00 (20.00~24.00)	0.317
Maximum pause and hypopnea time (s)	66.55 ± 15.32	64.48 ± 12.98	0.458
Total number of respiratory events (times)	181.50 (103.00~332.00)	134.00 (94.50~230.50)	**0.026**
Total time of respiratory event time (min)	52.50 (33.50~103.00)	48.00 (26.00~71.50)	0.147
Number of obstruction and hypopnea events (times)	178.00 (103.00~322.50)	129.00 (69.75~226.75)	**0.024**
Number of central respiratory events (times)	0.00 (0.00~2.00)	0.00 (0.00~0.00)	0.244
Number of mixed respiratory events (times)	3.00 (0.00~7.75)	2.00 (0.00~4.25)	0.225
Total proportion of the waking periods (%)	24.35 (14.03~34.25)	15.60 (11.25~26.00)	**0.030**
Total proportion of the REM periods (%)	17.18 ± 7.57	18.58 ± 7.19	0.365
Total proportion of the light sleep periods (%)	44.59 ± 12.75	50.08 ± 11.73	**0.034**
Total proportion of the deep sleep periods (%)	8.55 (4.70~14.55)	9.10 (4.55~15.45)	0.770
Average oxygen saturation (%)	93.55 (90.63~96.00)	96.05 (90.73~97.05)	**0.018**
Minimum oxygen saturation (%)	71.10 (60.93~83.03)	79.65 (66.25~88.48)	**0.027**
Oxygen depletion index (times/h)	31.00 (13.90~61.25)	20.10 (5.30~47.90)	0.076
Average heart rate (bpm)	66.50 (59.25~73.75)	66.00 (60.00~69.00)	0.590
Maximum heart rate (bpm)	100.00 (91.25~110.75)	99.00 (95.00~105.75)	0.699

The meaning of the bold values is p<0.05.

#### PSG Data of OSA Patients With ED and OSA Patients Without ED

The comparison of OSA with ED and OSA without ED revealed that sleep efficiency (p=0.036), average oxygen saturation (p=0.018) and minimum oxygen saturation (p= 0.027) were significantly lower in the OSA with ED group than in the OSA without ED group.

Moreover, the total number of respiratory events (p=0.026), number of obstruction and hypopnea events (p=0.024), total proportion of the waking periods (p=0.030) and total proportion of the light sleep periods (p=0.034) were significantly higher in the OSA with ED group than in the OSA without ED group (**Table 4** and **Figure 4B**).

#### ROC Curve Analysis of Related Factors in OSA Patients

As shown in **Table 4**, both OSA with ED and OSA without ED are closely related to the following 14 factors: BMI, neck circumference, waist circumference, hip circumference, ESS score, Beck depression score, ASEX score, sleep efficiency, average oxygen saturation, minimum oxygen saturation, total number of respiratory events, number of obstruction and hypopnea events, total proportion of the waking periods and total proportion of the light sleep periods.

**Figure 5:**
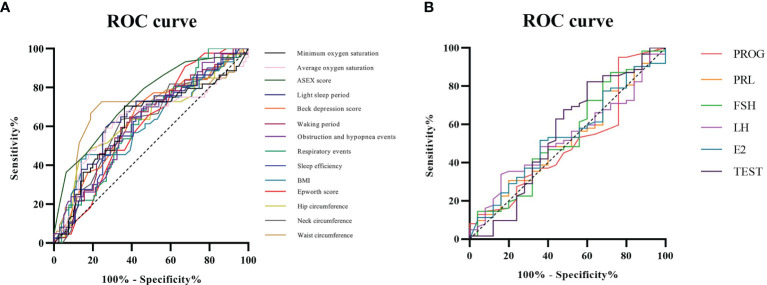
ROC curve analysis of related factors in OSA patients. **(A)** Compared with the IIEF-5 scoring standard, ASEX score and waist circumference had general diagnostic value, and the 12 related factors had low diagnostic value; **(B)** Compared with that of the IIEF-5 scoring standard, the diagnostic value of the six related factors was low.

ROC analysis showed that ASEX score had the highest AUC (area under the curve) at 0.738, followed in descending order by waist circumference (0.703), total proportion of the light sleep periods (0.651), Beck depression score (0.638), hip circumference (0.638), total proportion of the waking periods (0.630), average oxygen saturation (0.629), obstruction and hypopnea events (0.625), total respiratory events (0.624), neck circumference (0.622), minimum oxygen saturation (0.621), sleep efficiency (0.619), ESS score (0.612) and BMI (0.611).Compared with the IIEF-5 scoring standard, ASEX score and waist circumference had general diagnostic value, and the 12 related factors had low diagnostic value (**Figure 5A**).

#### The Relationship of the IIEF-5 Score With the AHI Value and ASEX Score in OSA Patients

**Figure 6:**
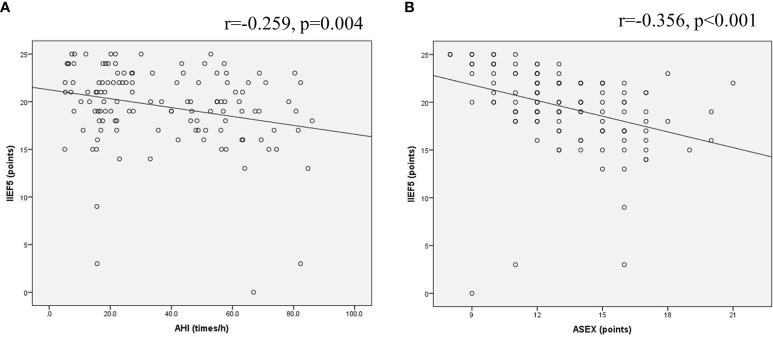
Scatter plot of the relationship of OSA patients. **(A)** The IIEF-5 scores of 124 OSA patients were negatively correlated with the AHI value; **(B)** The IIEF-5 scores were negatively correlated with the ASEX score.

The IIEF-5 scores of 124 OSA patients were negatively correlated with the AHI value, and the Pearson correlation coefficient was r=-0.259, P=0.004 (**Figure 6A**). The IIEF-5 scores were negatively correlated with the ASEX score, and the Pearson correlation coefficient was r=-0.356, P<0.001 (**Figure 6B**). The IIEF-5 score was not significantly correlated with any of the other items (P>0.05).

#### Sex Hormone Secretion in OSA With ED and OSA Without ED

A comparison of OSA with ED and OSA without ED showed that the secretion levels of PRL (p=0.793), FSH (p=0.599), LH (p=0.676), PROG (p=0.682) and TEST (p=0.431) were not significantly different between the groups (**Table 5** and **Figure 4C**).

**Table 5:** Sex hormone data of OSA with ED and OSA without ED.

Sex Hormones	OSA With ED (n=80)	OSA Without ED (n=44)	P
PRL (ng/mL)	19.08 (13.95~23.73)	18.74 (14.98~26.09)	0.793
FSH (mIU/mL)	4.81 (3.98~6.43)	5.10 (3.36~6.77)	0.599
LH (mIU/mL)	4.97 (3.80~6.77)	5.20 (4.05~6.28)	0.676
PROG (ng/mL)	0.23 ± 0.14	0.21 ± 0.11	0.533
E2(pg/mL)	18.76 ± 9.10	19.63 ± 8.52	0.682
TEST (ng/mL)	3.09 (2.53~3.87)	3.64 (2.31~4.68)	0.431

FSH, Follicle stimulating hormone; LH, luteinizing hormone; and TEST, testosterone; E2, estradiol; PROG, progesterone; and PRL, prolactin.

Moreover, ROC analysis showed that TEST had the highest AUC at 0.554, followed in descending order by FSH (0.536), E2 (0.535), LH (0.529), PRL (0.518) and PROG (0.504). Compared with that of the IIEF-5 scoring standard, the diagnostic value of the six related factors was low (**Figure 5B**).

#### Binary Logistic Regression Analysis of Related Factors in OSA With ED

According to clinical characteristics of OSA with ED, BMI (p=0.209), ESS score (p=0.232), Beck depression score (p=0.190), average oxygen saturation (p=0.349) and minimum oxygen saturation (p=0.257) were subjected to binary logistic regression analysis, and no significant difference was found between the groups (p>0.05), indicating that these factors were not independent influencing factors (**Table S1**).

### Treatment Response

**Figure 7:**
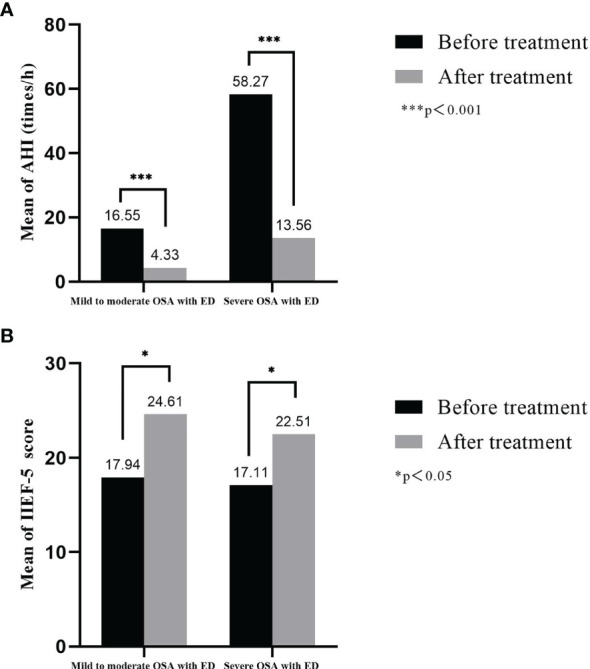
Treatment response of AHI and IIEF-5 score in OSA with ED. **(A)** Six months after UPPP surgical treatment, AHI values of patients with mild to moderate OSA and patients with severe OSA decreased; **(B)** The IIEF-5 of patients with mild to moderate OSA and patients with severe OSA score increased.

Fifteen patients with mild to moderate OSA with ED and fifteen patients with severe OSA with ED were followed up. Six months after UPPP surgical treatment, AHI values*** of patients with mild to moderate OSA decreased from 16.55 ± 5.60 times/h to 4.33 ± 1.23 times/h, and the IIEF-5* score increased from 17.94 ± 3.70 to 24.61 ± 4.76; the AHI values*** of patients with severe OSA decreased from 58.27 ± 14.10 times/h to 13.56 ± 7.25 times/h, and the IIEF-5* score increased from 17.11 ± 3.97 to 22.51 ± 5.37 (*p<0.05, ***p<0.001, **Figure 7**).

## Discussion

This study explores the relationship between OSA and ED, which is a relatively small area in the study of OSA clinical symptoms. It focuses on the erectile function status of patients with OSA and explores the clinical connection between the two diseases, which has high clinical value.

Patients who are diagnosed with OSA can receive psychological counseling and take corresponding intervention measures to reduce the general symptoms of OSA while improving the erectile function to achieve the cotreatment of OSA-related diseases.

### ED, Mainly Mild ED, Is a Common Symptom of OSA Patients

We found that ED was a common symptom of OSA patients and that symptoms were typically mild. The prevalence of OSA with ED was 64.52% (80/124), and the prevalence of severe OSA with ED was 73.02% (46/63). This figure is basically consistent with the following studies. Skoczynski (51) reported that the prevalence of ED in 61 Polish OSA patients was 72.1%; Popp (52) reported that the prevalence of ED in 246 German OSA patients was 65.0%; Budweiser (53) reported that the prevalence of ED in 91 German OSA patients was 61.5%; and Zhang (54) reported that the prevalence of ED in 207 Chinese OSA patients was 60.6% and that the prevalence of severe OSA with ED was 72.2%. Moreover, the prevalence of ED in 24 Turkish OSA patients was reported to be 54.2% by Gurbuz (55). Kellesarian (23) integrated several studies based on the IIEF questionnaire and found that the prevalence of OSA with ED was between 41% and 80%.

We also found that the factors associated with ED in OSA patients included the AHI value, age, BMI, ESS score, Beck depression score, sleep efficiency, total number of respiratory events, number of obstruction and hypopnea events, total proportion of the waking periods, total proportion of the light sleep periods, minimum oxygen saturation and average oxygen saturation. It is worth mentioning that the mechanisms related to these factors may be the causes of OSA with ED.

### The Prevalence of OSA With ED Increases With Age and BMI

ED obviously increases with age. Andersen (20) found that the prevalence of ED in young people (20-29 years old) was 7.3% and that the prevalence in elderly people (>60 years old) was 63.25%. According to Tufik (56), the 60- to 80-year-old population has 34.5-fold increased likelihood of having OSA compared with the 20- to 29-year-old population, and the prevalence of ED also doubles. This study compared the prevalence of OSA with ED in different age groups and found that the prevalence gradually increased with age (before the age of 50) and that the prevalence was as high as 70.59% (41-50 years old group), especially in the population with severe OSA, in which it was as high as 85.71% (18- to 30-year-old group). The prevalence of OSA with ED increases with increasing age. The prevalence of OSA in the 18- to 30-year-old group increased each year, and the prevalence of ED showed a 2.444-fold increase. The prevalence of combined ED increased by 2.800 times for OSA patients in the 31- to 40-year-old group with each 1-year increase in age; in the 41- to 50-year-old group, this prevalence increased by 3.400 times for OSA patients with each year. However, the prevalence of combined ED increased by 2.400 times for OSA patients in the 51- to 60-year-old group for each 1-year increase in age. Nevertheless, in the data for OSA with ED and OSA without ED, age did not show a significant difference between the groups (p=0.973), which may be related to the small number of people included.

The prevalence of OSA has increased with the increase in obesity worldwide, and obesity is closely related to ED (57). Peppard (58) found that weight gain is one of the most important risk factors for the increase in AHI values and the development of OSA, especially in patients with moderate to severe OSA. When weight increases by 10%, the progression of OSA increases 6-fold. This study found that the BMI of non-OSA subjects, patients with mild to moderate OSA, and patients with severe OSA increased with disease severity (p=0.001). A comparison of the prevalence of OSA with ED among different BMI groups showed that the prevalence of ED gradually increased with increasing BMI. Furthermore, the prevalence of ED among OSA patients increased with the increase in BMI value. For every increase of 1 kg/m^2^ in BMI of OSA patients, the prevalence of ED increased by 2.000 times in the normal weight group, by 2.471 times in the overweight group, and by 3.474 times in the obese group. In addition, OSA groups with and without ED, the BMI of patients in the OSA with ED group was significantly higher than that of patients in the OSA without ED group (BMI, 29.31 ± 4.08 kg/m2 vs. 27.72 ± 3.94 kg/m2, p=0.039).

### The Prevalence of OSA With ED Increases With The Severity of OSA

A study by Margel (59) showed that the severity of OSA (mainly the AHI value) may be related to the severity of ED. Skoczynski (51) suggested that OSA will lead to cardiovascular complications with age, leading to an increase in AHI values, and will be accompanied by increased dyspnea, which ultimately leads to the occurrence of ED Liu (60) reported abnormal circadian rhythm is associated with a decrease in sperm count, and as the circadian rhythm improves, the sperm count can be restored. Moreover, Vincent (61) reported that when OSA worsens, it causes fear of heart failure symptoms and promotes general anxiety about sexual activity. There are also studies with contrasting opinions. Santos (62) found that ED has a higher prevalence in OSA patients and that the severity of ED is related to age and diabetes but not to OSA itself. This study compared the prevalence of OSA with ED among different AHI groups and found that it gradually increased with increasing AHI values. The prevalence of mild OSA (AHI: 5-15 times/h) was at least 50%, and the prevalence was as high as 81.82% when AHI was ≥70 times/h. As the severity of OSA increases, the prevalence of ED increases; that is, for each 1 time/h increase in the AHI value of OSA patients, the prevalence of combined ED increased by 2.818 times. Each 1 time/h increase in the AHI value of patients with mild to moderate OSA increased the prevalence of combined ED by 2.259 times; moreover, each 1 time/h increase in the AHI value of patients with severe OSA increased the prevalence of combined ED by 3.706 times. However, in the OSA with ED and OSA without ED groups, the AHI value was not significantly different (p=0.102), which may be related to the small number of people included or the age difference of the included people.

Arousal and lack of sleep can have many effects on the normal physiology of the body, especially endocrine abnormalities and abnormal sympathetic nerve activity (63). Petersen (64) reported that chronic intermittent hypoxemia can reduce sexual activity and spontaneous erections in mice. Goh (65) suggested that the concentration of sex hormones is significantly related to sleep time. However, Stannek (21) reported that the severity of OSA may not be related to the severity of ED. This study found that the sleepiness score (ESS score) increased sequentially (p =0.003) among the non-OSA subjects, patients with mild to moderate OSA and patients with severe OSA and that sleep efficiency (p=0.036), minimum oxygen saturation (p=0.027) and average oxygen saturation (p=0.018) were significantly lower in the OSA with ED group than in the OSA without ED group. However, the total number of respiratory events (p=0.026), number of obstructive and hypopnea events (p=0.024), total proportion of the waking periods (p=0.030) and total proportion of the light sleep periods (p=0.034) were significantly higher.

Andersen (20) reported that the decrease in rapid eye movement (REM) sleep and the increased number of waking periods have a negative impact on the erectile function of male rats. Luboshitzky (66) found that during the first REM sleep episode, the level of testosterone secretion was the highest. Moreover, Liu (28) reported that factors such as reduced sleep in the REM periods can lead to damage to the peripheral nerves of the patient’s sexual organs, thereby causing ED. Furthermore, Gurbuz (55) found that an increase in the percentage of REM sleep has a negative impact on erectile function, while the total sleep time with a percentage of REM <20% seems to protect erectile function, but most of the research subjects were patients with psychological ED. Giles (67) showed that the REM period of the psychological ED group was longer than that of subjects without ED. This study found that the total proportion of the REM periods in the OSA with ED group was 17.18 ± 7.57%, while that in the OSA without ED group was 18.58 ± 7.19%, which was not significantly different (p=0.365) and may be related to the small number of people included.

### Improving Patients’ Anxiety and Depression Is Essential for the Treatment of OSA With ED

Papagiannopoulos (68) suggested that the contextual causes of psychological ED may include psychological distress (depression, job instability, and posttraumatic stress disorder), performance anxiety and partner-related difficulties (interpersonal instability). Zhang (54) reported that there is a well-known link between ED and mental illness and that depression and anxiety are the main psychological risk factors for ED in young men. Furthermore, Bandini (69) and Smith (70) found that depression is closely related to the severity of ED among men with ED who seek help. McCabe (71) reported that ED is related to the occurrence of depression and that treatment of ED with PDE5 inhibitors can improve depression symptoms. In a retrospective population survey of approximately 3,500 Finnish men between the ages of 18 and 48, Jern (72) found that depression is an important predictor of ED. In addition, the current study also showed that anxiety symptoms play an important role and that men with more sexual experiences suffer from ED less frequently. As mentioned earlier, anxiety may be involved in the pathogenesis of ED, and Caskurlu (73) suggested that it usually occurs at the beginning of sexual life. Zou (74) found that poorer sleep quality of college students correlated with mental health problems. This study found that the Beck anxiety scores of the non-OSA subjects, patients with mild to moderate OSA and patients with severe OSA increased with OSA severity (p<0.001). A comparison of OSA with ED and OSA without ED showed that the Beck depression score (p=0.011) of the OSA with ED group was higher than that of the OSA without ED group. However, the Beck anxiety score (p=0.367) was not significantly different between the groups, which may be related to the small sample size. Therefore, there may be a vicious circle mechanism among the depressive state (and the anxiety state), OSA and ED. In summary, improving the patient’s mental health is essential for the treatment of OSA.

### OSA With ED May Not Be Caused by Abnormal Levels of Sex Hormones

Corona (75) and Zhang (54) have shown that endocrine dysfunction, including changes in the hypothalamic pituitary axis, adrenal insufficiency, diabetes, hyperprolactinemia, hyperthyroidism and hypothyroidism, is involved in the progression of ED. OSA patients have repeated cycles of upper airway collapse and awakening, leading to sleep disturbances, sleep deprivation and fragmentation, which are manifested by intermittent hypoxia and reoxygenation. ED caused by intermittent hypoxia may have the following mechanisms: 1) vascular endothelial function damage (76); 2) oxidative stress (77); and 3) inhibition of sex hormone secretion (78), which mainly suppresses the response of central gonadal organs, reduces the levels of luteinizing hormone and testosterone, and affects libido (63, 79). Studies have also shown that elevated estradiol (80) and serum leptin (80–82) in obese patients can cause decreased testosterone secretion and ED. Zhang (54) and Luboshitzky (63) reported that the levels of testosterone, dehydroepiandrosterone sulfate, dehydroepiandrosterone and prolactin in the serum of patients with OSA was decreased, thereby reducing the bioavailability of testosterone and leading to ED. And Chen (31) found long and short sleep duration, as well as poor sleep quality, were linked to poor sperm quality metrics, even to ED.

There are still some studies that do not support the above view. Ludwig (83) reported that only 4% of men under the age of 50 have low levels of testosterone secretion but that it was not clear whether this is the cause of ED. Buvat (84), Hatzimouratidis (85) and Basar (86) reported that testosterone had the greatest effect on libido but not on ED. Soukhova (87) supported the above view with an animal model of intermittent hypoxia, which caused a decrease in libido in the animal model but did not change testosterone levels. Celec (88) found that long-term continuous positive airway pressure (CPAP) treatment had no significant effect on the levels of testosterone or estradiol in patients with OSA and pointed out that the positive effects of CPAP on sexual function reported by other studies may be caused by factors other than endocrine effects, which is consistent with the report of Onem (89). Moreover, Gambineri (90) suggested that this may be due to the low testosterone levels reported in previous studies and proposed that the relationship between testosterone and OSA is unrelated to obesity. Schiavi (91) analyzed other studies and showed that the increased risk of OSA and decreased testosterone levels actually depend on age as the main pathogenic factor. Interestingly, studies by Hammoud (92) and Barrett (93) do not support the view that OSA inhibits the secretion of testosterone and other sex hormones through the central nervous system. Liu’s study (94) showed that exogenous testosterone as a hormone replacement therapy can cause OSA. Even short-term treatment can aggravate sleep quality and OSA, but the mechanism is still unclear.

Killick (95) reported that testosterone had a limited therapeutic effect on OSA symptoms. The patient’s oxygen saturation index increased after 7 weeks, but there was no significant change in symptoms after 18 weeks of testosterone treatment. Furthermore, Mohammadi (41) recently studied testosterone secretion in 16 healthy controls and 39 OSA patients (10 cases of mild OSA, 16 cases of moderate OSA, and 13 cases of severe OSA). Notably, the control group and OSA patients had no significant differences in total testosterone levels or free testosterone levels (p>0.1). Jiang (96) examined sex hormone levels in 48 patients with OSA, of whom 23 had OSA with ED and 25 had OSA without ED. There was no significant difference in the secretion levels of estradiol (p=0.191), follicle-stimulating hormone (p=0.797), luteinizing hormone (p=0.412), prolactin (p=0.239), progesterone (p=0.964) or testosterone (p=0.07). Chen (30) reported semen and peripheral blood samples were taken from 656 male students in China for analysis of sperm quality and reproductive hormone levels. There was no link discovered between sleep duration and reproductive hormone.

This study summarized the sex hormone test data of the included population, and the results showed that the secretion levels of prolactin (p=0.728), follicle-stimulating hormone (p=0.062), luteinizing hormone (p= 0.294), progesterone (p=0.821), estrogen (p=0.686) and testosterone (p=0.056) were not significantly different among the control group, mild to moderate OSA group, and severe OSA group. Comparing OSA with ED and OSA without ED revealed that the secretion levels of prolactin (p=0.793), follicle-stimulating hormone (p=0.599), luteinizing hormone (p=0.676), progesterone (p=0.533), estrogen (p=0.682), and testosterone (p=0.431) in the OSA with ED group were not significantly different than those in the OSA without ED group. Compared with that of the IIEF-5 scoring standard, the sex hormone test had low diagnostic value. Therefore, it is speculated that OSA with ED may not be caused by abnormal levels of sex hormones but may be related to factors other than endocrine effects (such as obesity). Since free sex hormones are the main active form, it is recommended to use more advanced experimental equipment when studying sex hormones in the future to further distinguish between free and bound sex hormones.

### Research Limitations

This study has the following limitations, which need to be resolved in future studies. First, several studies (11, 29) indicate that erectile function may be related to a reduction in REM sleep in OSA patients. This study did not find a significant correlation with REM sleep in either the OSA with ED group or the OSA without ED group. In addition, there were only 11 non-OSA subjects in the group. In the future, it will be necessary to organize large-sample, multicenter clinical research to improve the scientific nature of the research. Second, the participants were all men who had been diagnosed with OSA, which may not be generalizable to other ED-related populations. Third, objective measurements (nighttime penile erection monitoring equipment and Doppler ultrasound equipment) can evaluate ED more reliably than questionnaire surveys (97–99), but related studies have also confirmed the effectiveness of IIEF questionnaires for ED evaluation (59, 97, 100, 101). The main basis of this study was the IIEF-5 questionnaire, and the secondary basis was the ASEX questionnaire. In future studies, the diagnosis of ED should include evaluation of potential cardiovascular risk factors and current use of related drugs, especially objective measures, to evaluate erectile function. Fourth, due to limited resources in this study, the levels of sex hormones were tested only once, but the level of hormone secretion in the human body may vary from day to night. Future research needs to collect data multiple times according to the circadian rhythm, distinguish between free and combined sex hormones, and further study the relationship between hormone levels and OSA. Finally, this study failed to further explore the biomolecular mechanism, and research centers need to continue to investigate animal models for experimental research.

## Conclusion

ED is a common symptom of OSA patients, and the symptoms are mainly mild. The prevalence of OSA with ED was 64.52%, and the prevalence of severe OSA with ED was 73.02%. The prevalence of OSA with ED increases with age, BMI, and AHI values. A mechanism related to the ESS score, sleep efficiency, total respiratory events, obstruction and hypopnea events, total waking periods, total light sleep periods, minimum blood oxygen saturation and average blood oxygen saturation may be the cause of ED in patients with OSA. Improving patient anxiety and depression is very important for the treatment of OSA with ED. The relationship between ED and sex hormone levels in OSA patients is still worthy of in-depth study. It is recommended that the free type and the combined type of sex hormones be further distinguished during testing, because the free type is the active form. Finally, UPPP surgical treatment is an effective treatment for OSA with ED. Its possible mechanism is protection of the peripheral nerves of the sex organs by improving nighttime hypoxia and arousal.

## Data Availability Statement

The original contributions presented in the study are included in the article/**Supplementary Material**. Further inquiries can be directed to the corresponding authors.

## Ethics Statement

The studies involving human participants were reviewed and approved by the Internal Review Board of the Institutional Ethics Committee of Shanghai General Hospital. The patients/participants provided their written informed consent to participate in this study.

## Author Contributions

CF and YY have contributed equally to this work. CF and YL designed the study. YY, RG, HL, and CL performed the collection and handling of the data. LC, YW, and CF analyzed the data and wrote the manuscript. YL and PD revised the manuscript. All authors discussed the data and accepted the final draft. All authors contributed to the article and approved the submitted version.

## Funding

This work was supported by the National Natural Science Foundation of Shandong Provincial (no. ZR2018MH017), the Key R&D Program Fund Project of Shandong Provincial (no. 2018GSF118001), the National Natural Science Foundation of China (no. 82072989) and the Fund Project of Shanghai Shen Kang Hospital Development Center (no.SHDC12020120).

## Conflict of Interest

The authors declare that the research was conducted in the absence of any commercial or financial relationships that could be construed as a potential conflict of interest.

## Publisher’s Note

All claims expressed in this article are solely those of the authors and do not necessarily represent those of their affiliated organizations, or those of the publisher, the editors and the reviewers. Any product that may be evaluated in this article, or claim that may be made by its manufacturer, is not guaranteed or endorsed by the publisher.

## Supplementary Material

Supplementary Figure S1 Distribution of IIEF-5 scores in ED patients and control patients. Summarizes the IIEF-5 score of the included population.
