# Supplementary figures and images for: Prevalence and Characteristics of Erectile Dysfunction in Obstructive Sleep Apnea Patients
Source: Front Endocrinol (Lausanne). 2022 Feb 18;13:812974. doi: 10.3389/fendo.2022.812974 (PMC8896119; doi:10.3389/fendo.2022.812974)

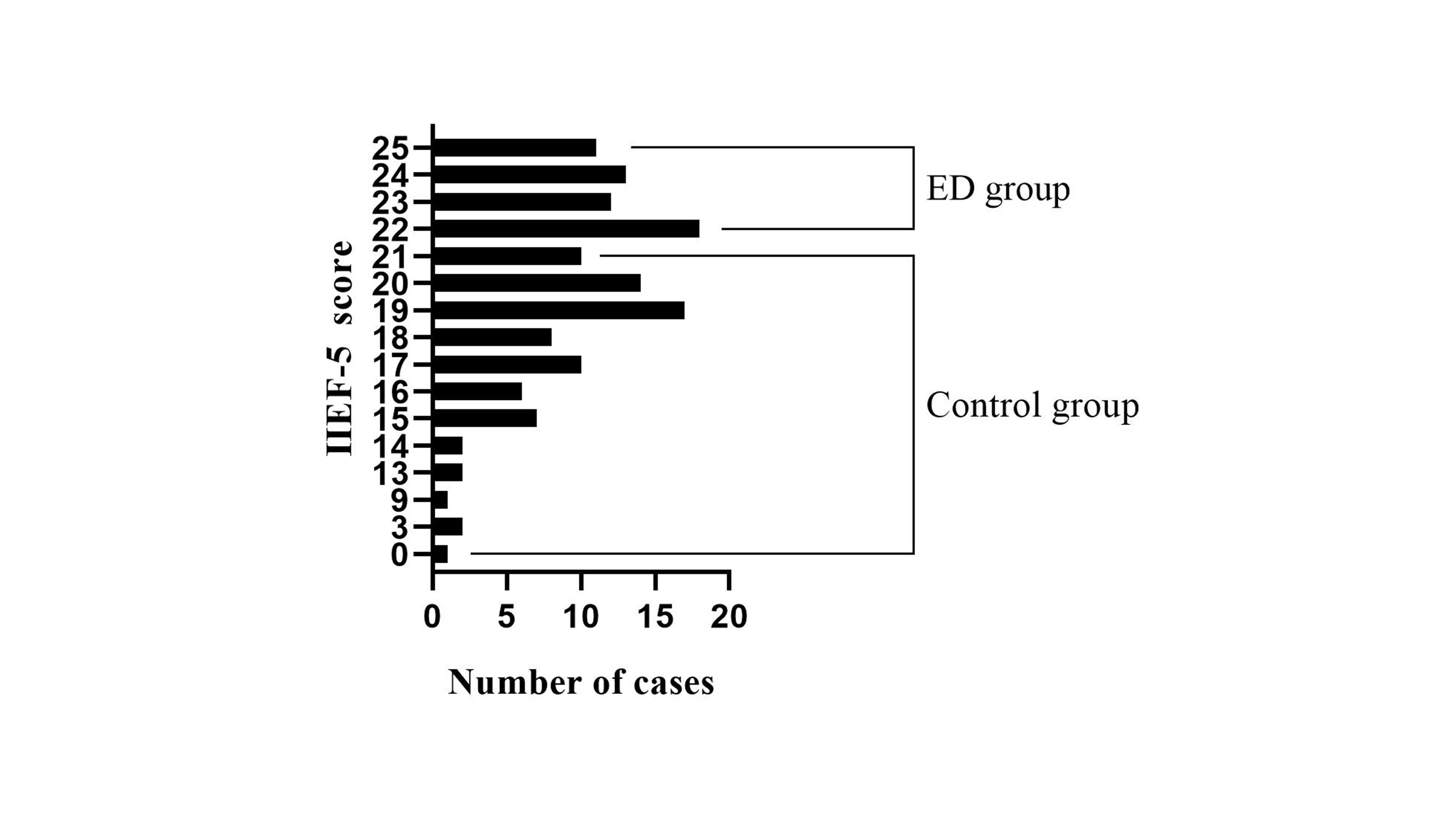

Supplement: Supplementary Figure S1 — Distribution of IIEF-5 scores in ED patients and control patients. Summarizes the IIEF-5 score of the included population. [file Image_1.tif]
