# Supplementary material for: Prevalence and Characteristics of Erectile Dysfunction in Obstructive Sleep Apnea Patients
Source: Front Endocrinol (Lausanne). 2022 Feb 18;13:812974. doi: 10.3389/fendo.2022.812974 (PMC8896119; doi:10.3389/fendo.2022.812974)
Supplement: Supplementary file 2 [file Table_1.pdf]

Table S1 Binary Logistic Regression Analysis of Related Factors in OSA with ED

Item	P	OR (95%CI)	OR (95%CI)
BMI (kg/m^2)	0.209	0.935(0.841-1.039)	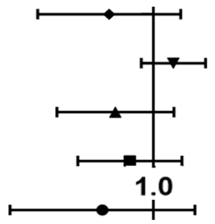
ESS score	0.232	1.027(0.983-1.072)	
Beck depression score	0.190	0.945(0.867-1.029)	
Average oxygen saturation (%)	0.349	0.965(0.896-1.040)	
Minimum oxygen saturation (%)	0.257	0.923(0.804-1.060)	
